# Enhancing Heart Rate Detection in Vehicular Settings Using FMCW Radar and SCR-Guided Signal Processing

**DOI:** 10.3390/s25185885

**Published:** 2025-09-20

**Authors:** Ashwini Kanakapura Sriranga, Qian Lu, Stewart Birrell

**Affiliations:** Centre for Future Transport and Cities, Coventry University, Coventry CV1 2TT, UK; kanakapura@uni.coventry.ac.uk (A.K.S.); ad5271@coventry.ac.uk (Q.L.)

**Keywords:** FMCW radar, signal processing, Signal-to-Clutter Ratio (SCR), in-cabin monitoring, contactless physiological monitoring

## Abstract

This paper presents an optimised signal processing framework for contactless physiological monitoring using Frequency Modulated Continuous Wave (FMCW) radar within automotive environments. This research focuses on enhancing heart rate (HR) and heart rate variability (HRV) detection from radar signals by integrating radar placement optimisation and advanced phase-based processing techniques. Optimal radar placement was evaluated through Signal-to-Clutter Ratio (SCR) analysis, conducted with multiple human participants in both laboratory and dynamic driving simulator experimental conditions, to determine the optimal in-vehicle location for signal acquisition. An effective processing pipeline was developed, incorporating background subtraction, range bin selection, bandpass filtering, and phase unwrapping. These techniques facilitated the reliable extraction of inter-beat intervals and heartbeat peaks from the phase signal without the need for contact-based sensors. The framework was evaluated using a Walabot FMCW radar module against ground truth HR signals, demonstrating consistent and repeatable results under baseline and mild motion conditions. In subsequent work, this framework was extended with deep learning methods, where radar-derived HR and HRV were benchmarked against research-grade ECG and achieved over 90% accuracy, further reinforcing the robustness and reliability of the approach. Together, these findings confirm that carefully guided radar positioning and robust signal processing can enable accurate and practical in-cabin physiological monitoring, offering a scalable solution for integration in future intelligent vehicle and driver monitoring systems.

## 1. Introduction

Contactless physiological monitoring has garnered significant attention across various domains, particularly in automotive environments where non-intrusive and contactless sensing solutions are preferred for occupant safety and health assessment. Among the technologies explored, Frequency Modulated Continuous Wave (FMCW) radar stands out due to its ability to detect very small physiological movements such as cardiac and respiratory signals without requiring physical contact. Unlike conventional sensors such as electrocardiograms (ECG) or photoplethysmography (PPG), which rely on skin contact and are often affected by placement constraints and user discomfort, FMCW radar provides a practical alternative by remotely sensing thoracic micro-movements induced by cardiac activity. However, implementing FMCW radar in vehicle cabins introduces a unique set of challenges. The interior environment is subject to complex background clutter from static structures, and motion artefacts arising from occupant movements and vehicle dynamics. These factors significantly degrade the signal-to-noise ratio and obscure the weak physiological signals of interest [1,2]. Therefore, robust signal processing techniques are essential to isolate and enhance these signals for accurate heart rate (HR) and heart rate variability (HRV) estimation. Conventional physiological sensors like ECG (Electrocardiography) and PPG (Photoplethysmography), while widely used, face significant limitations in dynamic vehicular environments. ECG is highly susceptible to motion artefacts and electrode misplacement [3,4], while PPG suffers from volumetric distortion and data loss due to movement [5]. These shortcomings necessitate the development of contactless alternatives capable of high-fidelity signal capture in non-static conditions [6].

Existing algorithms for radar-based HR estimation can be broadly categorised into two groups. The first group focuses on frequency-domain techniques, such as spectral peak analysis, matrix factorisation, and empirical mode decomposition, which are effective for HR but limited for HRV estimation [7,8]. The second group leverages time-domain or phase-based signal processing, which reconstructs a continuous waveform from which both HR and inter-beat intervals (IBIs) can be directly derived [9,10,11]. This latter approach is more suitable for HRV analysis and real-time monitoring. Recent studies have further demonstrated improvements in robustness through random body movement correction [12], posture-invariant HR estimation [13], and multi-occupant respiration tracking using time-division multiplexing [14]. Additional recent surveys, such as those conducted by Paterniani et al. [15], provide a broader context on radar-based vital sign monitoring. Accordingly, this research focuses on refining phase-based methods for enhanced accuracy and robustness under motion-afflicted conditions. Accordingly, this research focuses on refining phase-based methods for enhanced accuracy and robustness under motion-afflicted conditions. FMCW radars offer unique advantages over other radar types, such as IR-UWB (Impulse-Radio Ultra-Wideband), by balancing power efficiency, hardware simplicity, and effective range detection [16,17,18]. Unlike IR-UWB systems that rely on amplitude and suffer from increased ADC complexity, FMCW systems use phase information, making them better suited for non-invasive applications in constrained environments like vehicles. Advanced processing techniques, such as phase unwrapping and range bin selection, enable precise signal extraction even under cluttered and high noise conditions.

This paper presents an optimised signal processing framework tailored for contactless physiological monitoring using FMCW radar in automotive conditions. The proposed methodology integrates a set of signal enhancement strategies beginning with radar placement optimisation through Signal-to-Clutter Ratio (SCR) analysis. This is followed by a structured signal preprocessing pipeline comprising background subtraction to remove static reflections, range bin selection to localise chest motion [19], bandpass filtering to isolate cardiac frequency bands [9], and phase-based signal processing to derive precise physiological features. Phase unwrapping techniques are employed to resolve discontinuities in the radar phase signal, enabling accurate extraction of inter-beat intervals (IBI) [11]. Subsequent peak detection allows for the derivation of HR and HRV metrics. The performance of the proposed pipeline is validated in a controlled vehicle simulator environment, with ECG signals used as the ground truth for benchmarking. Experimental results confirm that the framework significantly improves physiological signal clarity and estimation accuracy despite the presence of motion artefacts and environmental noise. The present study focuses on optimising radar placement and developing a robust signal processing pipeline to extract HR and HRV from FMCW radar signals in laboratory and simulated driving conditions. This framework was later extended with deep learning methods and benchmarked against research-grade ECG, achieving over 90% accuracy in HR and HRV detection. While the current paper presents the foundational placement and signal processing work, the subsequent validation highlights the potential of this framework for reliable physiological monitoring in dynamic driving environments [20].

The key novelties of this study are as follows:

- Optimised Radar Placement via SCR Analysis: this work introduces a novel use of Signal-to-Clutter Ratio (SCR) analysis to identify optimal radar placement locations within the vehicle cabin, thereby maximising physiological signal quality and minimising background interference [21].
- Comprehensive Phase-Based Signal Processing Pipeline: the proposed framework integrates multiple processing stages—background subtraction, range bin selection, bandpass filtering, and phase unwrapping—resulting in precise extraction of heart rate (HR) and heart rate variability (HRV) from radar signals, despite motion-induced disturbances.

Validation in Realistic Vehicular Conditions: unlike prior work confined to controlled laboratory settings, this study conducts a validation within a dynamic vehicle driving simulator environment to establish practical efficacy, aligning with the gaps outlined in recent systematic reviews [6].

## 2. Materials and Methods

This section outlines the experimental procedures, radar configuration, and the signal processing techniques developed to extract heart rate (HR) and heart rate variability (HRV) using FMCW radar in controlled laboratory and simulated vehicular environments. All physiological signals were acquired exclusively from the radar, without using any reference sensors such as ECG.

### 2.1. FMCW Radar System and Placement Optimisation

The study employed the Walabot Developer Module, a short-range Frequency Modulated Continuous Wave (FMCW) radar system that uses a 2D antenna array and supports multi-directional scanning with high temporal resolution. The system operated over a frequency range of 3.3–10 GHz, yielding a theoretical range resolution of approximately 1.8 cm. The chirp delay was set to 250 ms with a chirp sampling frequency of 4 Hz. The maximum measurable range was 10 m, and the average transmitted power was −41 dBm/MHz, which complies with FCC safety guidelines. Data acquisition used up to 40 logical antenna pairs derived from 18 physical antennas, providing flexibility for MIMO-based sensing. The module was powered by a 5 V supply with a current consumption of 0.4–0.9 A.

To determine the most effective placement for physiological signal capture, a Signal-to-Clutter Ratio (SCR) analysis was conducted. SCR quantifies the strength of the desired physiological signal relative to background clutter [21,22] and is defined as

$$Signal\;to\;Clutter=\frac{P_{Signal}}{P_{Background\;Clutter}}$$

where P_signal_ denotes the average power of the radar signal captured in the presence of a subject, and P_Background Clutter_ is the power recorded under identical conditions without a subject present.

### 2.2. Laboratory-Based Experiment

**Figure 1:**
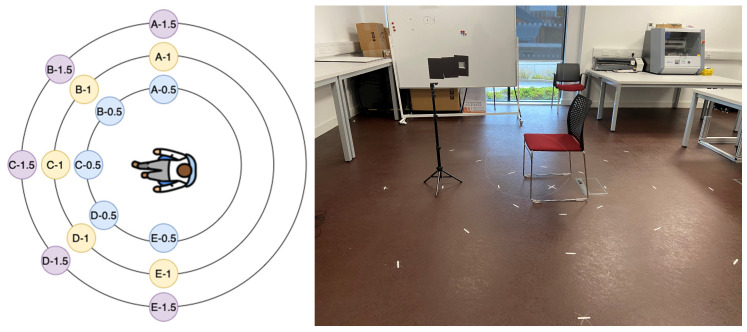
Radar placement—laboratory setting.

To account for potential variability in signal reception, five participants (three male and two female) were involved in the study. Although the participants’ height and weight were recorded for comparative reference in the event of discrepancies, the radar was consistently mounted at a height of 1 m to align with the upper thoracic region. Each subject was seated in a chair with the radar directly facing the chest. Data collection was carried out at three distances—0.5 m, 1 m, and 1.5 m—for each designated position (as illustrated in Figure 1).

**Figure 2:**
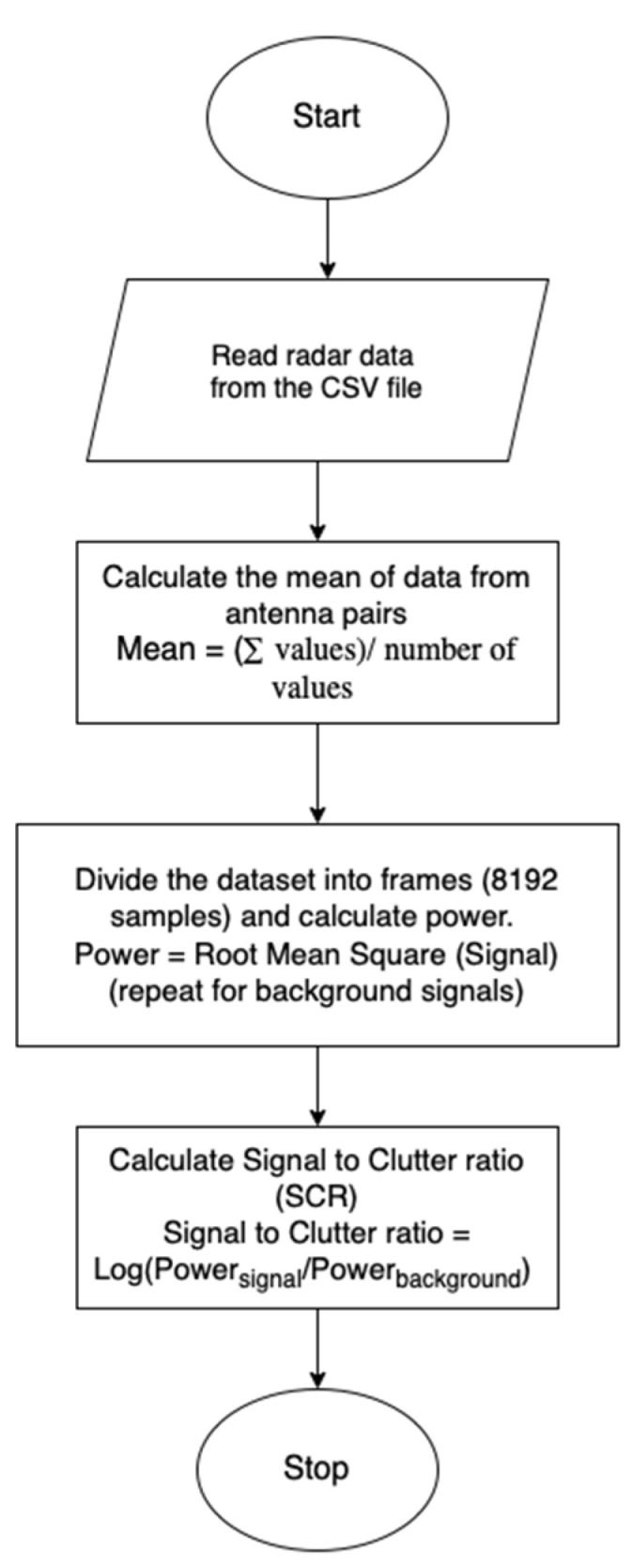
Radar data signal processing—Signal to Clutter Ratio (SCR) calculation.

To capture environmental background signals, a one-minute radar scan of the laboratory setting was conducted immediately following each participant’s session at every position. This scan served to record static environmental reflections, which were later subtracted from the participant data to isolate the physiological signal. The mean of 10 background frames was computed and subtracted from the participant signal frames to suppress background clutter [23]. Radar data were exported in CSV format for post-processing (as outlined in the signal processing flowchart, Figure 2. During acquisition, 40 logical antenna pairs were enabled, resulting in 40 channels of radar data per frame. For each radar position, both participant signals and corresponding background scans—each one minute in duration—were collected for analysis. At each position, intermediate frequency (IF) signals were collected over one minute, with and without a seated participant. Background recordings were used to calculate static clutter power, and a mean of ten background frames was subtracted from the active signal to isolate physiological components. Following this, a range FFT was applied to the IF signals to generate range–time maps. The range bin corresponding to the participant’s thoracic region was then selected, and the time-domain signal from this bin was used as the input for Signal-to-Clutter Ratio (SCR) evaluation. Signal-to-Clutter Ratio (SCR) values were later computed for each location based on this data. The flowchart of the signal acquisition is shown in Figure 2.

Preliminary results demonstrated that the radar positions at the 0.5 m radius—specifically positions 0.5B and 0.5C in Figure 1—exhibited the highest SCR values. Conversely, positions at 1.5 m consistently showed lower SCR, and 1.0 m positions displayed more variability. The findings indicated a clear correlation between proximity to the chest cavity and improved signal clarity, supporting the previous literature on radar placement for physiological monitoring.

### 2.3. Simulator-Based Validation

**Figure 3:**
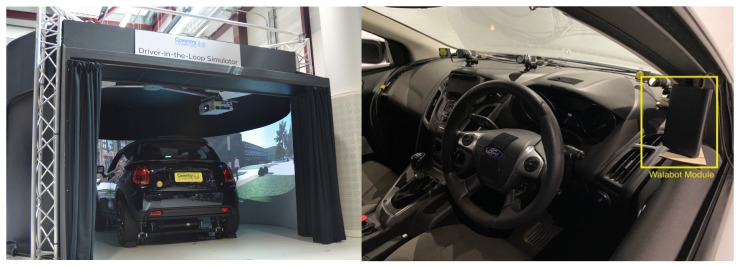
Radar data collection—Simulator Setup.

To substantiate the findings from the controlled laboratory environment, a second phase of the experiment was conducted in a high-fidelity driving simulator (as seen in Figure 3). The objective was to assess radar signal quality and validate optimal placement conditions under realistic vehicular settings. The experimental protocol mirrored the methodology used in the lab, with a focus on computing the Signal-to-Clutter Ratio (SCR) at various locations within the vehicle interior.

A total of five radar positions were evaluated based on practical constraints and prior placement literature:

- B-pillar on the driver’s side (Position 1),
- A-pillar on the driver’s side (Position 2),
- Behind the steering wheel (Position 3),
- Near the infotainment system (Position 4), and
- A-pillar on the passenger’s side (Position 5).

Each position was assessed for SCR using background and participant radar recordings. To maintain consistency and fairness in comparison, the distance from the radar to the driver was kept between 0.5 m to 1 m, except for Position 5, which ranged from 1.0 to 1.25 m due to spatial constraints within the vehicle cabin. Preliminary results suggested that the A-pillar on the driver’s side (position 2) produced consistently high SCR values and was selected for further physiological monitoring experiments. The signal acquired from Position 2 was chosen for further processing.

### 2.4. Signal Processing Framework

**Figure 4:**
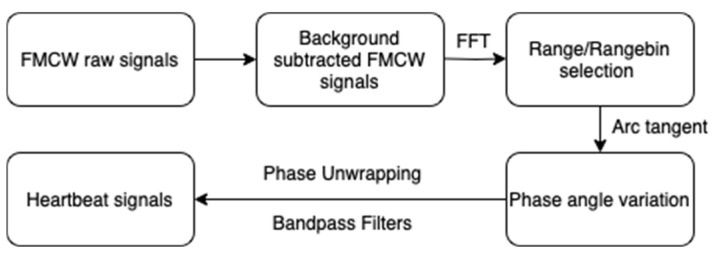
Designed pipeline to extract cardio-physiological signals from FMCW IF chirps [20].

Based on data from all five positions, with Position 2 (shown in Figure 3) being considered optimal, the radar data from Position 2 were processed through a structured pipeline designed to isolate and enhance cardio-physiological signals, as shown in Figure 4.

**Figure 5:**
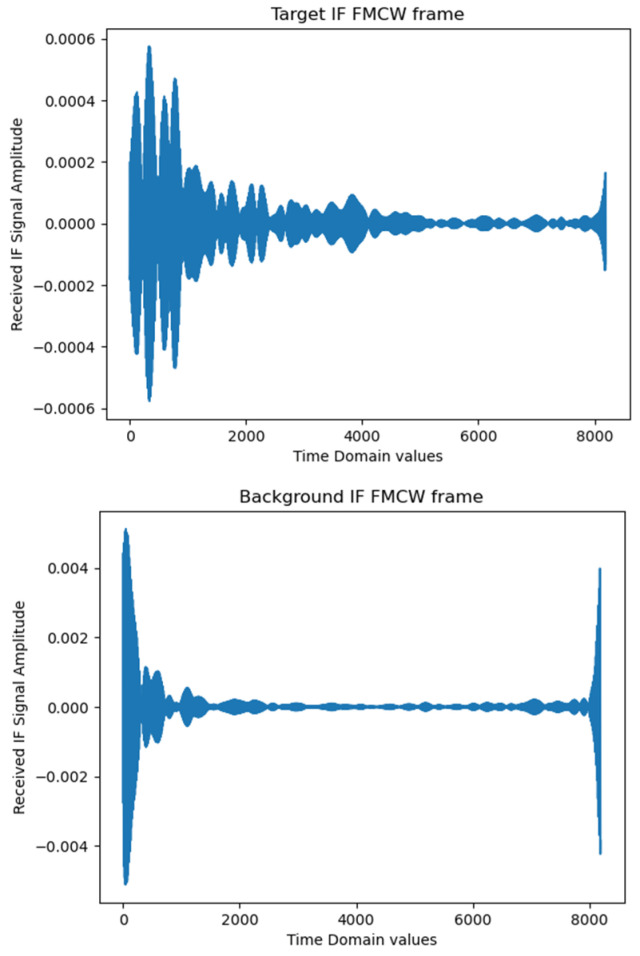
Raw FMCW IF signal—target and background frames (one frame—8192 values).

**Figure 6:**
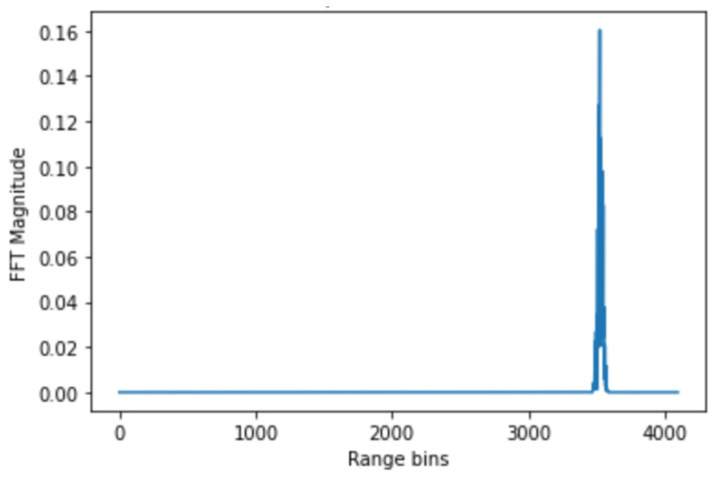
FFT spectrum of one frame in the FMCW signal.

The key steps are outlined below:

- Background Subtraction: Ten frames of background data, as seen in Figure 4 (recorded without the subject), were averaged to create a clutter reference. This reference was subtracted from each radar frame to suppress static environmental reflections and enhance signal-to-noise characteristics, see Figure 5.

- Range bin selection: For each radar frame, a Fast Fourier Transform (FFT) was performed along the range axis to produce a range profile (Figure 6). The FFT equation for range processing:

$$X\left(k\right)=\sum_{n=0}^{N-1}x\left(n\right)e^{-j2\pi kn/N}$$

Following the FFT range, the bin corresponding to the participant’s thoracic region was identified as the one exhibiting the highest amplitude variation. To ensure that this bin contained physiological activity, the phase signal was further examined for periodic modulation consistent with respiration and heartbeat. In both the laboratory and in-vehicle experiments, this selection was cross-checked against the known radar-to-chest distance to maintain consistency across participants.

- Bandpass filtering: A 4th-order Butterworth bandpass filter with cut-off frequencies between 0.8 and 2.0 Hz was applied to isolate the frequency range associated with cardiac activity. This suppressed both low-frequency respiration signals and high-frequency noise.
- Phase unwrapping: The selected range bin yields a complex signal comprising real R(t) and imaginary I(t) components. The instantaneous phase [11] ϕ(t) was computed as:

ϕ(t) = arctan [I(t)/R(t)]

**Figure 7:**
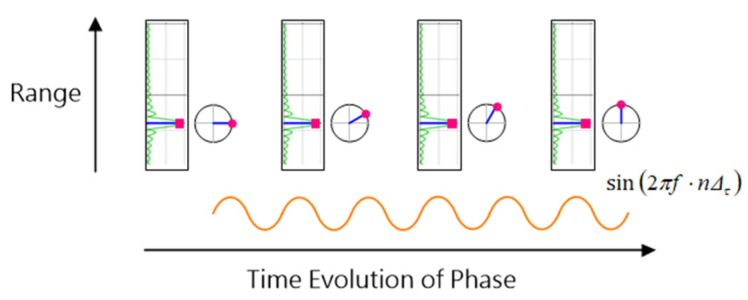
FFT spectrum illustrating range bins and the periodic phase modulation at the thoracic bin, which appears as a sinusoidal waveform over time.

Due to the cyclical nature of the arctangent function, phase discontinuities were corrected using a standard unwrapping algorithm to generate a continuous phase signal suitable for heartbeat detection (Figure 7).

**Figure 8:**
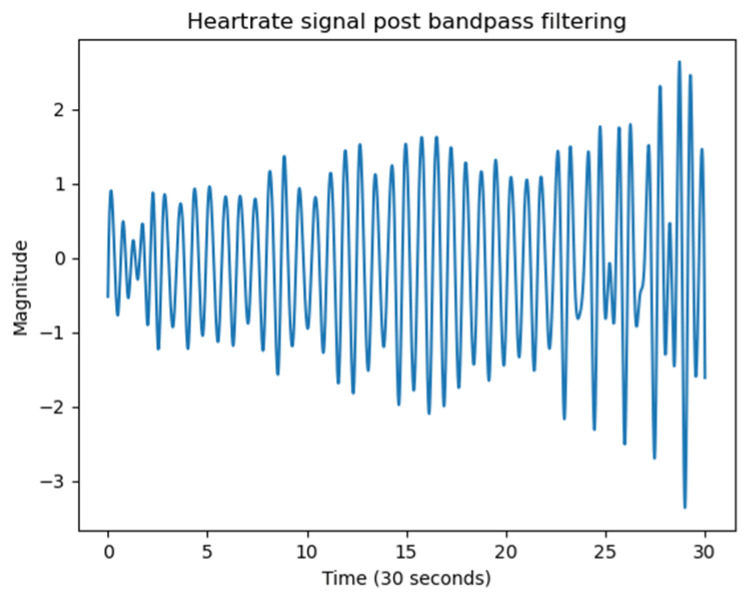
Phase-unwrapped and bandpass filtered (single antenna pair).

- Peak detection and HR/HRV estimation: Heartbeats were identified by detecting local maxima in the phase-unwrapped signal (Figure 8). The unwrapped phase signal includes contributions from both respiration and heartbeat. Given that the typical resting heart rate for adults lies between 60 and 100 bpm, a Butterworth bandpass filter with cut-off frequencies from 0.8 Hz to 2 Hz was applied to isolate the cardiac component. The time intervals between successive peaks were used to identify inter-beat intervals (IBIs).

The Butterworth bandpass filter description:

$$H\left(s\right)=\;\frac{1}{\sqrt{1+{(s/\omega_{c})}^{2n}}}$$

## 3. Results and Experimental Validation

This section presents the experimental outcomes of the study, which include signal quality assessments via Signal-to-Clutter Ratio (SCR) measurements in both laboratory and simulated vehicular environments, as well as qualitative validation of heart rate (HR) signals extracted through phase-based radar signal processing.

### 3.1. SCR Analysis in Laboratory Setting

**Figure 9:**
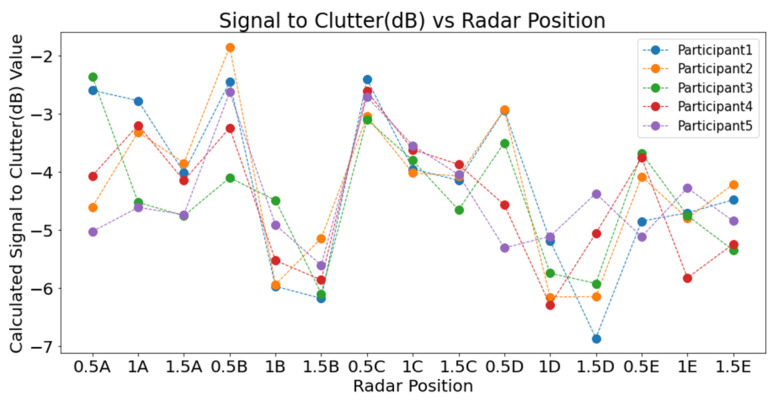
SCR (dB) versus positions for individual participants.

The first phase of validation involved a comprehensive laboratory experiment aimed at identifying optimal radar placement for physiological signal acquisition. SCR was computed across 15 radar positions, arranged in three concentric circles (radii of 0.5 m, 1.0 m, and 1.5 m) and five angular locations (A to E). To increase the validity and transferability of the results, each position was tested with five seated participants, and both background and target IF signals were recorded for one minute at 1 Hz. Results demonstrated that the radar positions at the 0.5 m radius, specifically positions 0.5B and 0.5C, exhibited the highest SCR values (Figure 9). Conversely, positions at 1.5 m consistently showed lower SCR, and 1.0 m positions displayed more variability. At position E, however, the SCR curve deviated from the expected distance–power trend due to partial obstruction from cabin geometry and a less favourable angle of incidence, which increased multipath clutter while reducing the effective chest echo power. The findings indicated a clear correlation between proximity to the chest cavity and improved signal clarity, supporting previous literature on radar placement for physiological monitoring.

**Figure 10:**
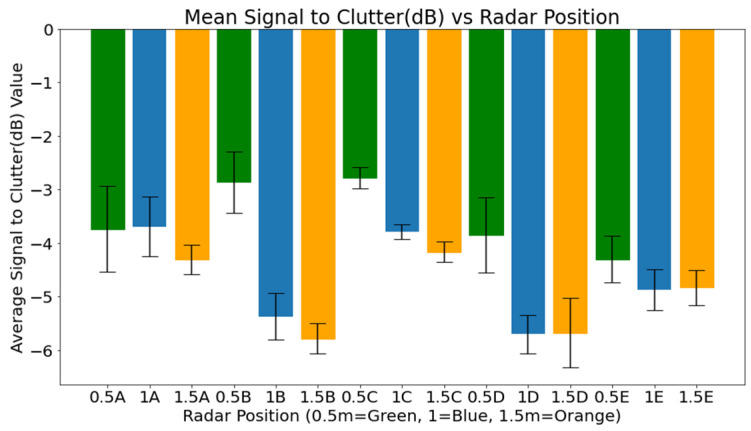
Mean Signal to Clutter (dB) versus positions.

As shown in Figure 10, SCR values at each radar position were averaged across participants to evaluate signal quality. Positions at a 0.5 m distance consistently yielded higher SCR values than those at 1.0 m and 1.5 m, confirming that closer proximity improves signal detection. Among these, positions 0.5B and 0.5C recorded the highest values. In contrast, the lowest SCR values were found at 1.5 m across all angular points. The 1.0 m positions showed mixed results, with 1A and 1C performing better than others at that distance. These outcomes highlight the importance of proximity in optimising radar placement for physiological monitoring.

### 3.2. SCR Analysis in Vehicle Setting

**Figure 11:**
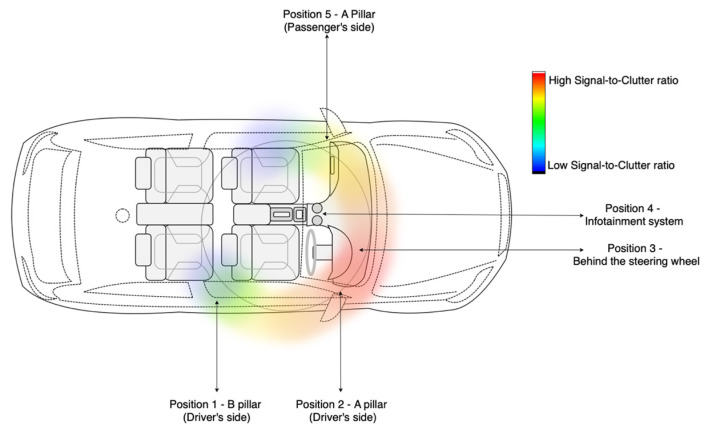
Heatmap of Signal to Clutter values in different positions in the car.

The radar placement experiment was repeated within a high-fidelity vehicle simulator (Figure 9) to further validate the laboratory findings under more realistic driving conditions. Five radar positions were evaluated: B-pillar (driver side), A-pillar (driver side), behind the steering wheel, near the infotainment unit, and A-pillar (passenger side). The A-pillar on the driver’s side (position 2) produced consistently high SCR values and was selected for further physiological monitoring experiments. A heatmap visualisation of SCR across cabin positions further validated this choice (Figure 11).

### 3.3. Physiological Signal Processing Outcomes

**Figure 12:**
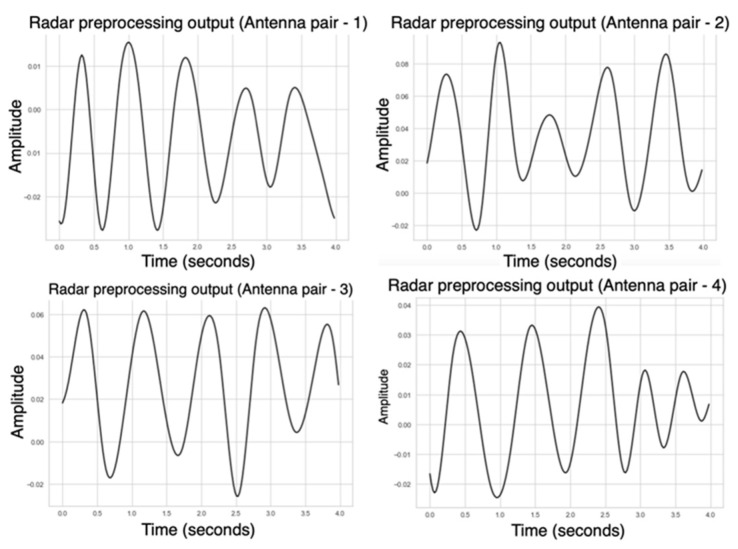
Representative phase output derived from acquired radar measurements after signal pre-processing (background subtraction, range bin selection, filtering, and phase unwrapping).

Following placement optimisation, radar signals were processed using the proposed pipeline. Range bin selection, background subtraction, and bandpass filtering were applied to isolate the physiological signal. Phase unwrapping of the complex signal revealed periodic waveforms with clearly identifiable peaks corresponding to heartbeats. During the laboratory experiments, all 40 logical antenna pairs were activated. Following this phase, we realised that the required sampling rate for cardiac activity was lower than initially assumed. According to the Nyquist theorem, reliable detection of heart rate within the 0.8–2 Hz range (48–120 bpm) only requires sampling at least twice the maximum frequency of interest. Based on this observation, in the driving simulator experiments, we activated only four antenna pairs, which provided sufficient sampling while reducing data volume and computational load. Figure 12 illustrates a representative phase output, where a regular series of peaks was observed, corresponding to heartbeat events. These peaks were identified using a peak detection algorithm, and the inter-beat intervals (IBIs) were computed to extract heart rate features. Despite the presence of minor phase shifts across different antenna pairs, the overall signal structure supported reliable HR detection and provided a strong foundation for later benchmarking studies.

## 4. Discussion

The experimental results of this study demonstrate that radar placement is a critical determinant of signal quality for contactless physiological monitoring in automotive environments. Through a detailed Signal-to-Clutter Ratio (SCR) analysis, it was confirmed that radar positions closer to the subject, specifically those aligned with the upper torso, yielded significantly higher SCR values. This finding was consistent across both laboratory and vehicle simulator environments. Positions such as the A-pillar on the driver’s side offered high SCR and practical feasibility without obstructing the driver’s view, thus making it an ideal location for in-vehicle deployment.

The phase-based signal processing framework developed in this research effectively extracted heartbeat signals from radar data by integrating background subtraction, range bin selection, bandpass filtering, and phase unwrapping. The resulting phase signals exhibited periodic structures suitable for inter-beat interval (IBI) estimation. The framework developed in this study provided consistent cardiac waveforms under mild motion conditions. In subsequent work, this pipeline was extended with LSTM-based deep learning and validated against research-grade ECG, where radar-derived HR and HRV achieved over 90% accuracy [20]. These complementary results reinforce the robustness of the approach presented here and demonstrate its ability to support both conventional signal processing and machine learning-based applications.

Several challenges were also observed. Motion artefacts, particularly from limb and torso movements, introduced noise that sometimes overlapped with the cardiac frequency band. Although the controlled vehicle simulator did not replicate the full range of vehicular dynamics, voluntary movements were encouraged to simulate real-world conditions. The observed phase signals remained sufficiently robust, highlighting the effectiveness of the signal processing pipeline. However, in real-world driving scenarios, additional noise suppression strategies may be required.

Furthermore, hardware-related constraints such as phase noise and thermal noise posed inherent limitations to signal clarity. While such noise sources are typically addressed through hardware enhancements, this study focused on software-based preprocessing techniques due to practical limitations. Nonetheless, the results affirm that a well-optimised signal processing pipeline can significantly mitigate the impact of environmental and system-induced noise.

## 5. Conclusions

This study presented a fully non-contact radar-based approach for the extraction of physiological signals within vehicle interiors, emphasising both radar placement optimisation and robust signal processing. The following key conclusions were drawn:

- The proposed signal processing pipeline, comprising background subtraction, range bin selection, filtering, and phase unwrapping, enabled the accurate extraction of cardiac-related signals from raw FMCW radar data.
- A novel radar placement methodology based on SCR analysis identified the A-pillar (main structural pillar off-centre to the driver) as the optimal location for in-cabin physiological monitoring.
- This study demonstrated the feasibility of radar placement optimisation and a structured signal processing framework for extracting HR and HRV in vehicular environments. The framework was subsequently extended and benchmarked against research-grade ECG with LSTM-based modelling, achieving over 90% accuracy [20]. Together, these findings validate the reliability of the radar-derived signals and highlight the scalability of the approach for future in-cabin driver monitoring applications.

This experimental research confirms the feasibility of using FMCW radar for in-cabin contactless HR monitoring and offers a validated signal processing architecture adaptable to real-time applications. Future work may focus on extending this framework with adaptive filtering techniques, higher sampling rates, and multi-modal sensor fusion to enhance robustness under dynamic vehicular conditions.

## Data Availability

Data are contained within the article.
